# New trends in biomedical engineering and bioinformatics applied to biomedicine - special issue of IWBBIO 2014

**DOI:** 10.1186/1475-925X-14-S2-I1

**Published:** 2015-08-13

**Authors:** Franscisco M Ortuño, Carolina Torres, Peter Glösekötter, Ignacio Rojas

**Affiliations:** 1 Department of Computer Architecture and Computer Technology, CITIC-UGR, University of Granada, 18071 Granada, Spain; 2 Department of Biochemistry and Molecular Biology, University of Granada, 18071 Granada, Spain; 3 Department of Electrical Engineering and Computer Sciences, Muenster University of Applied Sciences, 48565 Steinfurt, Germany

## 

*From *2nd International Work-Conference on Bioinformatics and Biomedical Engineering - IWBBIO 2014 Granada, Spain. 7-9 April 2014

The present supplement in the BioMedical Engineering OnLine journal is composed by 7 extended papers from the original papers selected from the 2^nd^ International Work-Conference on Bioinformatics and Biomedical Engineering (IWBBIO 2014) held in Granada (Spain) during April 7-9, 2014. These papers were selected due to their significant relevance and the importance of the presented researches and the obtained outcomes.

IWBBIO 2014 aimed to create a friendly environment where scientists, engineers, educators and students could discuss and present their latest ideas, researches, theories, models and applications. This conference was specifically oriented to interdisciplinary and multidisciplinary fields encompassing disciplines such as biomedical engineering, computer science, mathematics, statistics, biomedicine or bioinformatics. The IWBBIO 2014 also sought to establish and to strengthen the cooperation and collaboration among scientists through the presentations of their more recent researches and works in high-quality original papers (including work-in-progress). These original researches dealt with, among others, important challenges in fields like genomics, proteomics, next generation sequencing (NGS), high performance bioinformatics, biological systems modelling, medical informatics, e-health, computational healthcare, biomedical engineering or telemedicine.

The IWBBIO 2014 has counted on 197 papers that were carefully reviewed and accordingly accepted by several referees (each submission was reviewed by at least 2, and on the average 2.8, program committee members or additional reviewers). As shown in the conference proceedings of IWBBIO 2014 [1], both full contributions and abstracts were accepted for presentation in this conference. Nevertheless, it can be appreciated that a large majority of the contributions were presented as full papers.

It is also important to note that IWBBIO 2014 was held as a two-track conference containing a total of 21 oral and 2 poster sessions. The final number of attendees was 213, from 49 different nationalities. The countries which have contributed with more researchers are (in this order): Spain, Italy, Poland, United States, France, United Kingdom, Brazil, India, Germany and China.

From these contributions, a reduced number of authors were then invited to extend their conference papers and to submit them to this supplement in BioMedical Engineering OnLine. The selection was done taking into consideration the evaluation and opinion of reviewers, chairs of the different sessions and guest editors. In this regard, the present supplement has been considered to be specially focused on different ideas applied to the improvement of healthcare and biomedical engineering: from novel strategies for the diagnosis and treatment of severe diseases to the implementation of e-health frameworks to make easier the data management in the biomedical environment. Following, a brief introduction of each selected article is provided for the sake of consistency and readability of the present supplement.

The first paper authored by Prof. Arthur Williams et al. [2] essentially proposes the design of two symptom check-lists for adults and children (TRSC and TRSC-C, respectively) with oncological diseases. These lists aim to facilitate the identification of therapy-related symptoms produced in these patients, to reassure patients by making more understandable these symptoms and to improve the patient-clinician communications. These checklists are indeed being incorporated as a pilot experience in different hospitals and they have been adapted to several languages and cultures.

The paper by Cristina Callau et al. [3] describes two different approaches for quantifying the CK19 marker in breast cancer tissues. The proposed algorithms are based on computer vision techniques for digital images of tissues to automatically perform the immunohistochemistry (IHC) quantification. These two methods are implemented to avoid errors in the visual quantification of expert pathologists. Outcomes in this study have shown a reasonable concordance between the quantification performed by the two automated methods and those ones provided by the pathologists. Thus, these findings could be a promising beginning for the future implementation of an automated prognosis tool.

Daniel Passeri et al. [4] address in their paper a numerical model to simulate the structure of Ion Sensitive Field Effect Transistors (ISFETs) in biosensors by using 3D-TCAD tools. This simulation successfully models the electrolyte environment as an ad-hoc parametric layer in the semiconductor, allowing its direct incorporation into FET. It also provides a significant improvement in the technology design by reducing time and costs thanks to implemented simplifications such as larger dimensions or less number of target charge sites.

Following, the paper by Razvan Ghinea et al. [5] presents a group of 402 predictive models to estimate the reflectance spectrum in biomaterials, more specifically, in dental resin composites. Each of the proposed models corresponds to a reflectance factor and they were based on a Multiple Non-Linear Regression (MNLR) algorithm to determine the spectrum of 49 different experimental dental resin composites. Thus, after a training step, the predictive models have shown an average error <3.5% reaching values lower than the theoretical acceptability threshold in the materials or even smaller than the perceptibility threshold.

In the next paper, Franciszek Binczyk et al. [6] propose an adaptive algorithm and other numerical solutions to reduce the phase error in five different algorithms applied in Nuclear Magnetic Resonance (NMI) spectroscopy. The optimization of the phase correction was then validated by using both *in silico *and phantom data achieving an accuracy of 99.5%. Thus, the obtained results may help to determine more accurate diagnosis.

The paper by Oresti Baños et al. [7] describes an open source Android framework to acquire, handle, store and visualize sensor data in mHealth mobile applications. More specifically, authors present a software environment properly designed to make easier and faster the development of medical health applications as well as the usage of sensors included in smartphones, tablets and wearable devices. Additionally, the paper shows the so-called mHealthDroid, a particular app to collect and visualize health data which have been implemented by the presented framework.

Finally, Vanathi Gopalakrishnan et al. [8] present a novel methodology to classify pediatric cardiomyopathies by means of information extracted from Magnetic Resonance Images (MRI). In particular, Bayesian rules are implemented to analyze 83 patients and controls and to extract the biomarkers associated to the disease. The accuracy and area under curve (AUC) results obtained for the proposed dataset and a cross-validation procedure allows to interpret that this tool could contribute to the assessment of MRI biomarkers and, therefore, to enhance the diagnosis and prognosis of these cardiomyopathies. Moreover, authors confirm the proposed methodology is flexible and extensible, being also applicable to other cardiovascular diseases.

As Guest Editors, we would like to sincerely acknowledge all the authors for their high quality contributions as well as the expert referees for their useful suggestions and comments during this review procedure. We would also like to express our sincere gratitude to Profs. Kenneth R. Foster and Fong-Chin Su, Editors-in-Chief of BioMedical Engineering Online, for giving the opportunity to publish this supplement. Finally, we are very thankful to Jennifer Egar, Assistant Project Manager for Supplements of BioMed Central, for the constant assistance with the edition and publication of this supplement. It has been a pleasure for us to collaborate with all of you. We finally invite all authors and interested readers of this supplement to participate in future IWBBIO conferences, which will be announced at http://iwbbio.ugr.es.

## Competing interests

The authors of this introductory article and the guest editors of the special issue declare that they have no competing interests.

## Declarations

Publication costs for this article were funded by the Regional Government of Andalusia under grant agreement P12-TIC 2082.

## Notes

This article has been published as part of *BioMedical Engineering OnLine *Volume 14 Supplement 2, 2015: Select articles from the 2nd International Work-Conference on Bioinformatics and Biomedical Engineering (IWBBIO 2014). The full contents of the supplement are available online at http://www.biomedical-engineering-online.com/supplements/14/S2.
